# The unsung women of citriculture: bridging science and society through macro and micro analysis for improved technology adoption in citrus value chain

**DOI:** 10.3389/fsoc.2025.1656780

**Published:** 2025-10-14

**Authors:** Sangeeta Bhattacharyya, Deepa Lal, Arnab Roy, Priyanka Lal, Pinaki Roy, Samrat Sikdar, Suchandra Dutta, Tannishtha Bardhan

**Affiliations:** ^1^Agricultural Extension, ICAR-Central Citrus Research Institute, Nagpur, Maharashtra, India; ^2^Horticulture, Krishi Vigyan Kendra, ICAR- Central Institute for Cotton Research, Nagpur, Maharashtra, India; ^3^Agricultural Economics, ICAR-Indian Agricultural Statistics Research Institute, New Delhi, India; ^4^Agricultural Economics, ICAR-Indian Institute of Pulses Research, Kanpur, Uttar Pradesh, India; ^5^Krishi Vigyan Kendra, Sitamarhi, Bihar, India; ^6^School of Human Sciences, College of Agriculture and Life Sciences, Mississippi State University, Starkville, MS, United States; ^7^Faculty of Agriculture and Rural Development, Ramakrishna Mission Vivekananda Educational Research Institute, Kolkata, West Bengal, India; ^8^International Cooperation Division, Ministry of Agriculture and Farmers’ Welfare, New Delhi, India

**Keywords:** women, citrus value chain, sustainable citriculture, gender gap, science and society, macro and microanalysis, technology gap, women empowerment strategies

## Abstract

**Introduction:**

Women constitute 43% of the global agricultural workforce, and remain largely undocumented in several sectors, especially citrus farming (citriculture). Although citrus fruits are cultivated commercially in more than 150 countries, significant gender disparities exist in citriculture, such as women facing drudgery, lack of access to resources, advisories, technical information, and opportunities for capacity development.

**Methods:**

In this study, the quantum of documentation done in “science” on “women in citriculture” was analyzed through “macroanalysis” whereby bibliographic assessment and systematic review of literature of 306 articles ranging from 1929 to 2024 available in “gender and citrus farming” from 35 major citrus producing countries was done using the Preferred Reporting Items for Systematic Reviews and Meta-Analyses (PRISMA) guidelines and Rayyan Qatar Computing Research Institute (QCRI). A cross-country visualization of gender disparities affecting agricultural technology adoption by women farmers was also done. For validating the findings with “society,” a “microanalysis” based on the theoretical grounding of Kabeer’s empowerment framework was conducted by interviewing 300 women citrus growers of Nagpur, India, on three dimensions of empowerment, namely, resources, agency, and achievements in the context of technology adoption.

**Results:**

The macroanalysis revealed 10 major gender disparities of 3 categories on a longitudinal and cross-sectional scale. Only 20% of respondents had a high Technology Adoption Score (4–6). Furthermore, respondents with sound technical know-how of citriculture were found to face lesser degrees of socio-personal gender disparities (*t*-value = 2.02, significant at the 0.05 level) than those who lacked technical knowledge.

**Discussion:**

Systematic strategies for women empowerment, based on Gender Transformative (GT) approaches, were outlined through macroanalysis. These strategies aim to enhance the impact of agricultural technologies, improve livelihoods, and promote sustainable citriculture and social development in line with the achievement of Sustainable Development Goals at microlevel. Developmental Goals achievement of Sustainable Development Goals at microlevel.

## Introduction

The world population is expected to reach 9.1 billion by 2050, necessitating a 70% increase in overall food production (FAO, 2009) to feed the teeming billions. This challenge rests on the shoulders of the global farming community. Agricultural development and above all the empowerment of “primary food producers” or the “farmers”; need to be the topmost priority of nations. At the same time, the global agricultural system suffers from a lack of quality scientific data to evaluate the effectiveness of existing commitments or policies, or to use as a basis for decisions on marketing, investments, or policies (World Bank, 2010). In other words, the agricultural “science” is suffering due to the inadequacy of documentation.

In the already persistent data void, the cornerstone of the global farming community, that is, the “women in agriculture,” remain as “invisible farmers” even to date (Velde et al., 2020). Women constitute 43% of the global agricultural labor force, even after owning less than 15% of the land (Babu and Gajanan, 2022). Women also face discrimination in livestock ownership, equal pay, decision-making, access to credit and financial services, with additional labor of caregiving in the farm household. Her labor remains largely unacknowledged, considered unimportant, ignored, and, most of the time, undocumented. In this backdrop, the one sector of horticulture where women’s role remains largely “untold” or undocumented is perennial fruit farming, in which citrus is a major fruit crop cultivated widely across several nations of the world. Citrus fruits are cultivated commercially in more than 150 countries of the world and rank second in production in the world, accounting for 161.8 million tons produced in more than 10.2 million hectares in 2021 (Gonzatto and Santos, 2023).

### Women in citriculture: the unsung in citrus science

In citrus cultivation, or “Citriculture,” women have long been associated with citrus production, processing, packaging, and marketing. The citrus industry (pre- and post-production and marketing) provides livelihood to a significant number of women workforce all over the world. However, there is no aggregated data available on the number of women’s engagements. Since the 1890s, women have comprised the majority of packing house labor in California (Maier, 2024). In India, which is the third largest citrus producer in the world, the participation of women was more in the marketing of lemon than in production and decision-making aspects (Sahu et al., 2018). There are significant gender disparities in citrus farming globally, with women facing drudgery, pay disparity, lack of access to land ownership, resources, critical inputs, technical information, and opportunities for capacity development. However, a simple search on the internet can reveal the glaring inadequacy of scientific literature, statistical databases, documents, books, or anything written on the topic of “women in citriculture” across the world to date.

### Conceptual and theoretical framework

The inadequacy of scientific research on women in citriculture made the authors conceptualize this study to assess the quantity of research and documentation (science) that has actually been done on women in citriculture, to date, across the globe using the “macro” level (longitudinal and cross-sectional research) research approach (https://saylordotorg.github.io/text_principles-of-sociological-inquiry-qualitative-and-quantitative-methods/s05-01-micro-meso-and-macro-approache.html) (github, n.d.). To statistically analyze the data available in public domain related to involvement of women in citrus value chain, a descriptive analysis of bibliographical data, systematic review was done and gender disparities enlisted. The study aimed to identify the gender disparities that affect citrus-based technology adoption by women farmers. For validation of macro findings, the “microlevel” research approach (https://saylordotorg.github.io/) (github, n.d.) was adopted to rank the enlisted gender disparities by women citrus growers of Nagpur district of Maharashtra, India, in August 2024. The effect of gender disparities on citrus-based technology adoption by women farmers was then studied in a microanalysis as well. The Gender Transformative (GT) approaches were explored to bridge science and society at the community level. Thereafter, subtle strategies for women empowerment were outlined to improve technology adoption by women in citriculture.

For microanalysis, a theoretical framework was required to construct the data collection instrument. It is a well-known fact that, despite the critical roles women play across agricultural value chains, including citriculture, their access to resources, decision-making authority, and technical capacity remains disproportionately low (Doss, 2018; Meinzen-Dick et al., 2019). These structural inequalities not only limit women’s productivity but also hinder broader development outcomes such as food security and sustainable farming practices. To analyze these gendered disparities in a structured and multidimensional way, the microanalysis section of this study derived its theoretical grounding from Kabeer’s empowerment framework (Kabeer, 1999). It stems from the understanding that women empowerment is about the process by which those who have been denied the ability to make strategic life choices acquire such an ability. According to Kabeer (1999), the ability to exercise choice incorporates three interrelated dimensions: resources (access and future claims to material, human, and social resources); agency (processes of decision-making); and achievements (wellbeing outcomes). Empowerment is the interrelationship of these three dimensions. Agency is central to empowerment, that is, how a choice is made and put into effect by women in a household; resources are the medium through which agency is exercised, and achievements are outcomes of the agency. Kabeer later emphasized that each of the three resources implied by the indicators—access to education, access to paid work, and political representation—is essential to achieving gender equality and women empowerment (Kabeer, 2005). Drawing on the above theoretical ground, the microanalysis part of this study was formulated to explore the access of women in citriculture to resources like land holding, education, paid work (agricultural or non-agricultural) or unpaid labor; the agency which they command like control over farm and non-farm income, decision-making ability and authority in household, ease of technological adoption, technical knowledge of citrus farming/processing and their achievements like membership in Self-Help Groups (SHGs), contact with extension workers, participation in skill development programs on citrus farming/processing. The data from these dimensions would also reveal the extent of gender disparities that the women respondents were facing.

**Figure 1:**
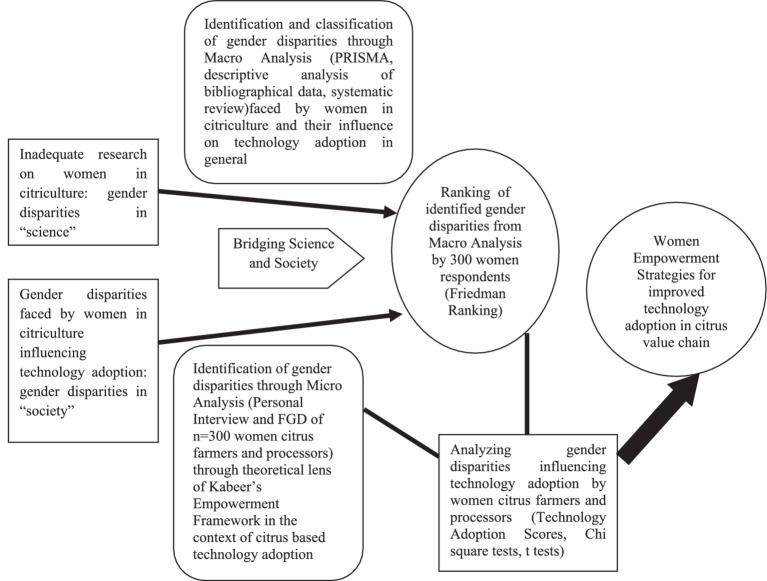
Conceptual framework of the study.

Thus, the study was conceptualized (Figure 1) to identify both through macro- and microanalysis, the gender disparities faced by women citrus farmers and processors, the technology adoption level of women respondents, the categories of disparities that influence their technology adoption, and accordingly outline women empowerment strategies for improved technology adoption in the citrus value chain.

## Materials and methods

### Location of the study and sampling plan for microanalysis

**Figure 2:**
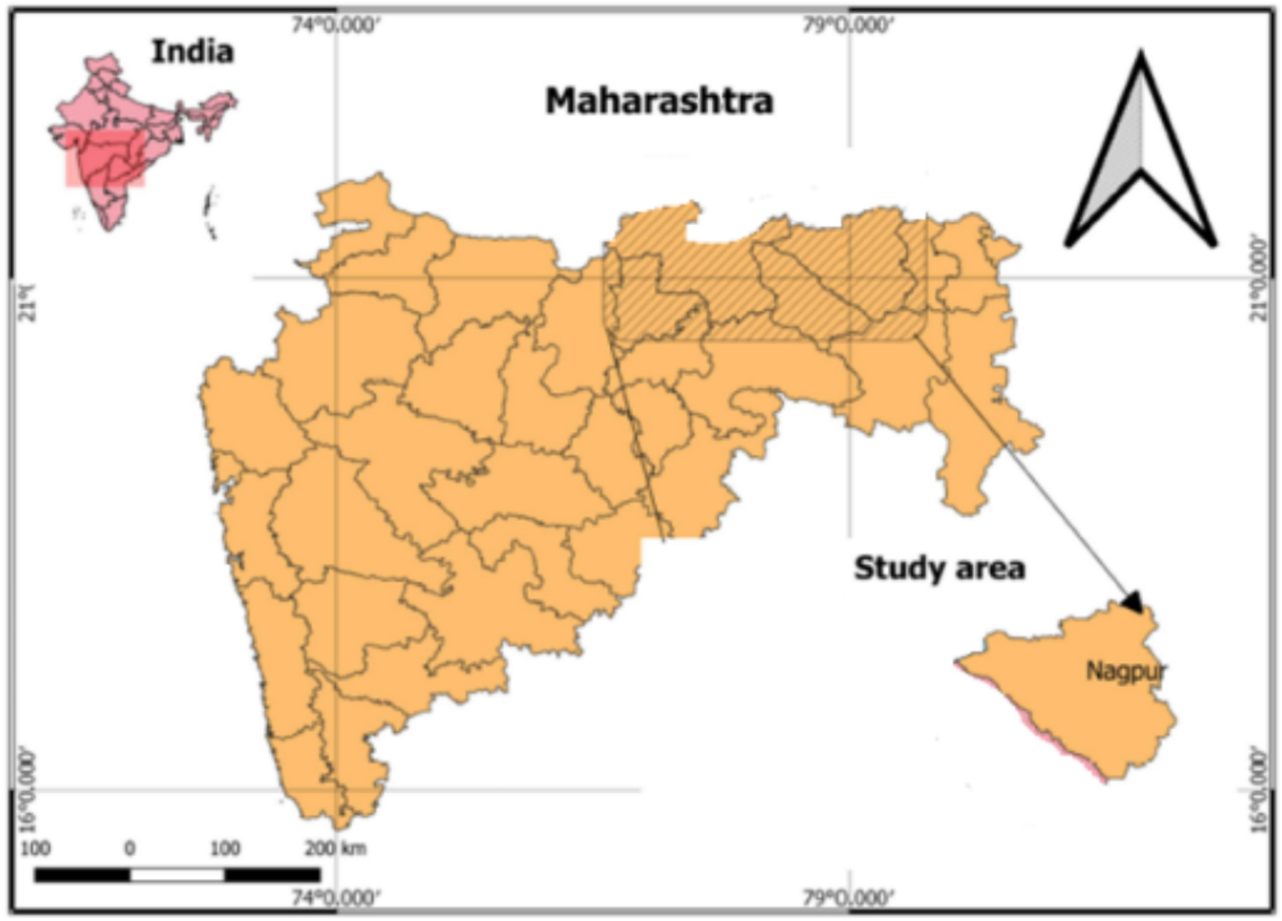
Location of microanalysis: Nagpur, India.

For assessing the presence or status of “women of citriculture” in “society,” “microlevel” analysis was done. India is the third largest producer of citrus in the world and the largest producer of limes and lemons specifically (Ministry of Agriculture and Farmers Welfare, 2023). The globally renowned GI-tagged Nagpur mandarin (*Citrus reticulata* Blanco) is named after the Nagpur district of Maharashtra state, India. Accordingly, Nagpur (Figure 2) was purposively selected for conducting the microanalysis. A sample of 300 women (*n* = 300) who were directly or indirectly engaged with citrus farming or processing (making value-added citrus products) and belonged to citrus farming households was selected purposively for the study from two geographical locations, namely, Beltarodi village of Nagpur Rural block of Nagpur district and Sonegaon village of Kalmeshwar block of Nagpur district. The respondents were members of various Self-Help Groups (SHGs) of Nagpur district. The concentration of SHGs is maximum in these two blocks; hence, purposive selection of respondents was done from SHGs of these two regions. Therefore, 150 respondents each from two locations were selected.

### Research design and method of data collection for microanalysis

The *ex post facto* research design was followed. A semi-structured interview schedule was developed. The respondents were personally interviewed, and data were collected through Focus Group Discussions (FGDs).

### Interview schedule for microanalysis

The schedule (Table 1) included questions about gender issues and their effect on technology adoption in citrus farming and processing among women perceived through the lens of Kabeer’s empowerment framework. The content validity and construct validity of the questions/items were checked through a relevancy test during the development of the schedule. Content validity of this variable was ensured by incorporating questions/items about three dimensions of Kabeer’s empowerment framework (Kabeer, 1999, 2005). Construct validity was ensured through expert consultation. The questionnaire was sent to 100 experts in sociology, gender, and rural development. Out of 22 items, only 18 items had a relevancy score above 0.80. Hence, these 18 items (Parts I–III) were included in the final schedule. The schedule was also pre-tested with non-sample respondents in a pilot study before final data collection.

**Table 1:** Interview schedule for microanalysis: Gender disparities and technology adoption through the lens of Kabeer’s empowerment framework.

Part I: Resources profile	
1.	Name, age, address, and phone number
2.	Land holding in their own name (a. Yes; b. No)
3.	Size (in hectares (ha)) of citrus orchard (irrespective of its own name or not)
4.	Educational qualification
5.	Primary Engagement (a. Farming; b. Other job; c. Housewife)
6.	If your primary engagement is farming, does it involve unpaid or paid work?

Part II: Agency profile	
1.	What activities are you involved in within the citrus value chain (farming/processing/marketing)?
2.	Do you have any say in your household in deciding which technology to adopt in the farming/processing of citrus? (a. Yes, I decide = 3; b. We jointly decide with husband/father = 2; c. I give inputs and my husband/father takes the final decision = 1; d. My husband/father does not consult me = 0)
3.	Who has control over the farm or non-farm income of the household? (a. Yes, I have = 3; b. We jointly have with husband/father = 2; c. I partially have, but my husband/father takes the final decision = 1; d. My husband/father does not allow me to control the income = 0)
4.	Who has control over your earnings from farm or non-farm activities? (a. Yes, I have = 3; b. We jointly have with husband/father = 2; c. I partially have, but my husband/father takes the final decision = 1; d. My husband/father does not allow me to control the income = 0)
5.	Who makes major decisions on the healthcare, education, etc. of children or other family members? (a. Yes, I decide = 3; b. We jointly decide with husband/father = 2; c. I give inputs and my husband/father takes the final decision = 1; d. My husband/father does not consult me = 0)
6.	Do you face domestic violence? (Yes = 0; Sometimes = 1; No = 2)
7.	Are you free to move out of your house for necessary activities without seeking anyone’s permission or depending on anyone? (Yes = 0; Sometimes = 1; No = 2)

Part III: Achievements profile	
1.	Do you think you have sound technical know-how of citrus farming/processing? (Yes = 1; No = 0)
2.	Do you have any access to extension or any kind of citrus advisory services? (Yes = 1; No = 0)
3.	Have you received any kind of skill development training from horticulture/agriculture institutes for your involvement in the citrus value chain? (Yes = 1;s No = 0)
4.	Are you part of any Self-Help Group (SHG), Farmer Interest Group (FIG), Farmer Producer Organization (FPO), Farmer Producer Company (FPC)? (Yes = 1; No = 0)
5.	Are you a representative/worker of any political party? (Yes = 1; No = 0)

Part IV: Correlating macro- and microanalysis	
1.	Rank the enlisted gender disparities (from most severe = 1 to least severe = 10) you face as a woman, which hinder your contribution to the citrus value chain?

### Influence of gender gap on technology adoption gap

Questions 2 of Part II and questions 1–3 of Part III of the interview schedules (Table 1) were designed to tap the decision-making power or role of women in citrus-based technology adoption. If a respondent scores maximum in all three questions, she will score 6. The respondents were then classified into categories according to their obtained Technology Adoption Scores. The analysis was done to reveal the influence of the gender gap on the technology adoption gap in citrus.

### Search strategy for macroanalysis

For “macrolevel” analysis, the Preferred Reporting Items for Systematic Reviews and Meta-Analyses (PRISMA) guidelines were used to review the scientific literature available on “women in citriculture” (Liberati et al., 2009). An exhaustive literature search was conducted of all peer-reviewed literature that focused on women and gender dimensions of the citrus industry across the world. That included all studies published until 30 June 2024 using a combination of search terms given in Table 2. Seven electronic platforms—ISS Web of Science, Scopus, EconLit, JSTOR, Science Direct, and EBSCO—were searched. Separate searches were conducted manually on other platforms such as Google Scholar, AgEcon and repositories of organizations such as the Consultative Group on International Agriculture Research (CGIAR), United Nations Food and Agriculture Organization (FAO), World Bank, International Food Policy Research Institute (IFPRI), International Fund for Agricultural Development (IFAD) and Indian Council of Agricultural Research (ICAR) which are closely linked with global and Indian agriculture. A Boolean search approach was followed for searching the databases by combining all the search terms and phrases in a group with the “OR” operator and the entire search groups with “AND” operator. As per the requirements of the database, the search query was adjusted. After the search process, the citations were fed into Rayyan QCRI, a web and mobile application that supports the initial screening of abstracts and titles through semi-automation.

**Table 2:** Electronic database search terms for gender disparities in citriculture.

Theme	Search terms
Citrus	Citrus, lemon, mandarin, orange, and lime
Women and gender	Women, women farmers, smallholder farmers, women in gender, women in agriculture, agricultural activity, women’s time, time use, the role of women, empowerment of women, level of participation of women, gender gaps, and gender dynamics
Location	Any country
Thematic area	Agriculture

### Inclusion criteria for macroanalysis

**Figure 3:**
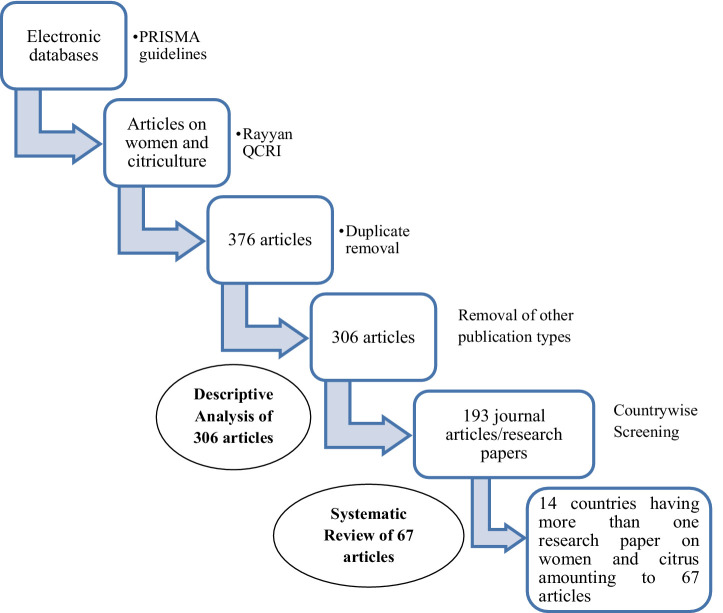
Flowchart of macroanalysis.

The search resulted in 376 articles. After removing duplicates, a total of 306 articles were included in the descriptive analysis. A significant number of other publication types were also found. Hence, for the systematic review, a total of 193 journal articles that had the keywords of “citrus” and “women.” or “gender” were initially included. However, after further screening of the country-wise articles, it was found that only 14 countries had published more than one research article in journals based on the above-mentioned keywords. The final result was 67 articles from 14 countries dated between 1970 and 2024 composed of keywords, namely, citrus, women, and gender, were subjected to systematic full text review (Figure 3) for identification/enlisting of gender gaps in the global citrus sector and possible strategies to bridge the gender gaps. Only countries with multiple research articles were selected to maximize the scope of the systematic review, ensuring the accurate assessment of women’s involvement in these countries.

### Cross-country visualization of gender gap influencing technology gap

The similar methodology of PRISMA was adopted for conducting a systematic review of research studies conducted across the world on the theme of gender gaps affecting agricultural technology adoption by women farmers. As specific studies on citricultural technology adoption by women were not available, studies about agricultural technology adoption were included. As the first systematic review pertained only to gender disparities faced by women in citriculture, the second systematic review (Table 3) on gender gaps influencing the technology gap was deemed essential to capture a glimpse of how gender issues hinder technology adoption in agriculture as a whole.

**Table 3:** Electronic database search terms for gender gaps influencing technology adoption in agriculture.

Theme	Search terms
Women and gender	Women, women farmers, smallholder farmers, women in gender, women in agriculture, agricultural activity, women’s time, time use, the role of women, empowerment of women, level of participation of women, gender gaps, gender dynamics, and gender issues
Adoption of technology	Adoption, technology, agricultural technology, adoption rate, adoption behavior, extent of adoption, technology gap, factors of adoption, and determinants of adoption
Location	Any country
Thematic area	Agriculture

The search across seven electronic platforms yielded 1,054 research studies, of which 996 articles remained after duplication was removed. Since the objective of this analysis was not to depict the inadequacy of research in this topic but to highlight the gender gap’s influence on the technology adoption behavior of women farmers across nations, only a systematic review was conducted, and the descriptive analysis of bibliographical data was avoided. However, for a systematic review of full texts, studies from countries with the highest number of research studies on women in citriculture were selected to assess the adoption behavior of women in agriculture in those countries.

### Validating macrolevel gender disparities at microlevel

The gender disparities identified through macroanalysis were ranked by respondents in microlevel analysis. The disparities faced by women citrus growers were ranked according to the value of the mean rank. The disparity with the highest mean rank was considered as the most vital constraint for women citrus growers in contributing to the citrus value chain.

Furthermore, an independent sample *t*-test was conducted to find out if there was a significant difference in the degree of disparities faced by women citrus growers who had sound technical know-how of citrus farming or processing, and those who lacked sound technical knowledge.

## Results and discussion

### Presence of “women in citriculture” in “science”: macroanalysis

**Figure 4:**
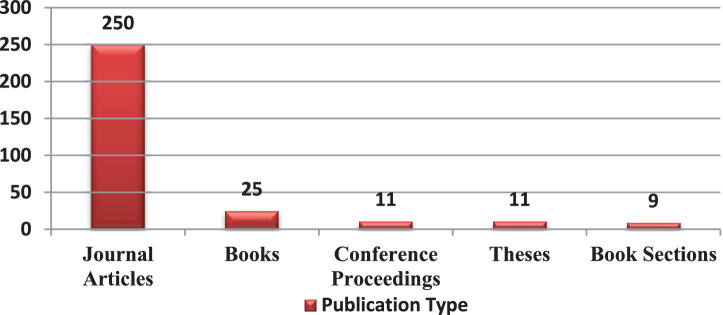
Categories of publication having “citrus” and “women” keywords.

During the review (assessment of “science”) of scientific literature, the majority of publications were found to be journal articles (250), followed by 25 books related to citrus cultivation in specific countries that mentioned women farmers. There were also conference proceedings, theses and book sections (Figure 4).

A close examination of the publication ranges reveals that citriculture and women’s participation in it were documented as early as 1929, albeit not extensively. Mostly around the 1950s and 1970s, the documentation picked up. The inclusion of this theme in mainstream scholarly research, that is, in theses, was done as late as 2007. To assess the consistency in research on the theme, it was found that one author from Pakistan consistently published four articles on citriculture, mentioning women farmers in each of them. Eight authors consistently published three or more articles related to the search terms “citrus” and “women.”

**Figure 5:**
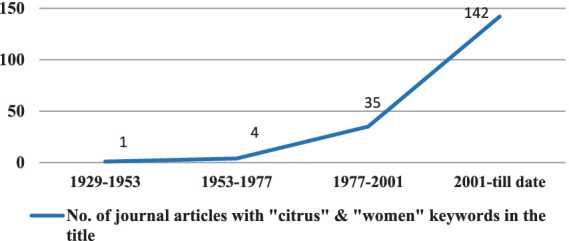
Increasing trend of journal articles with titles having “citrus” and “women” keywords.

After further screening of the 306 articles, only journal articles (250) were considered for further analysis. Among journal articles, only those articles that had the keywords “citrus” and “women” mentioned in their titles were selected to analyze the trend of documentation of this topic. A total of 182 such articles were found (Figure 5).

In the first 24 years, 1929–1953, there was only one article published. The number increased to four between 1953 and 1977 and to 35 between 1977 and 2001. From 2001 to the present date, 142 articles have been published, and the trend can be considered increasing over the years as per the trend graph (Figure 5).

**Figure 6:**
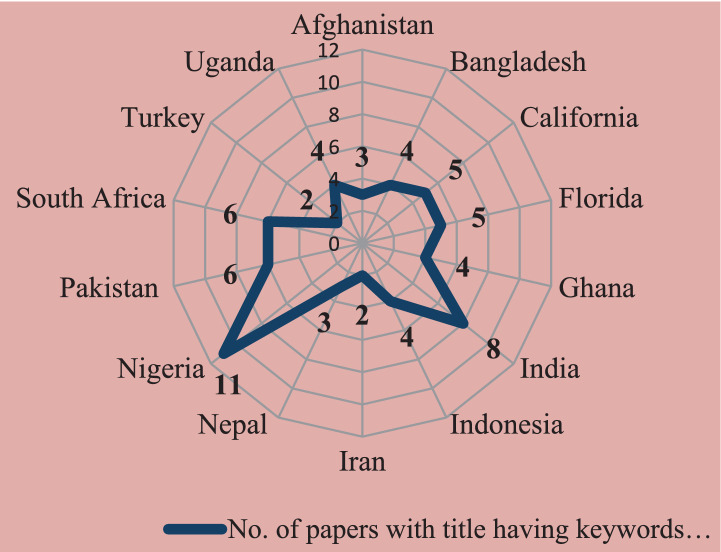
Countries with more than one research article having keywords “citrus” and “women” in their titles.

The 182 articles that were shortlisted originated from 35 countries, and most of them were from the regions of sub-Saharan Africa and South Africa, but a considerable amount of documentation has been done in India, Pakistan, and some other Asian countries too (Figure 6).

Among them, a total of 14 countries had research articles with the title having keywords “citrus” and “women” (Figure 6). Among them, Nigeria had the highest number of research articles (11), India the second highest (8), while Pakistan and South Africa (6 each). A total of 67 articles originated from these 14 nations. Thus, it is evident that gender issues in citriculture have been discussed in science and research of developing countries mostly.

### Systematic review of “science”: identification and classification of gender disparities

**Figure 7:**
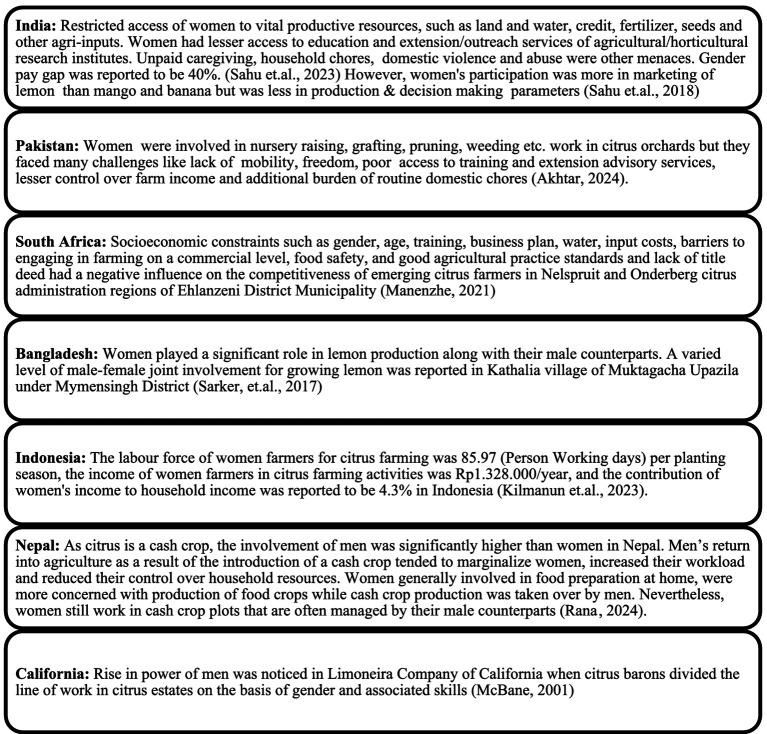
Systematic Review of ‘Science’ on “women in citriculture.”

A final systematic review was done for macroanalysis using the full texts of 67 articles from the 14 countries. Country-wise gender disparities were reported (Sahu et al., 2023; Sahu et al., 2018; Akhtar et al., 2024; Manenzhe, 2021; Sarker et al., 2017; Kilmanun et al., 2023; Rana, 2024; McBane, 2001) (Figure 7).

The descriptive analysis of literature revealed a handful of research/science studies done on women in citriculture. The systematic review further revealed the gender disparities existing in the citrus value chain at a global level and, accordingly, the issues were classified into three broad heads of socio-personal, economic, and technical disparities with sub-heads under each (Table 4).

**Table 4:** Classification of gender disparities in citriculture identified through macroanalysis.

Sl. No.	Category of disparities	Gender disparities in citrus value chain identified through macroanalysis
A	Socio-personal disparities	Sole responsibility of taking care of the family and children
		Low decision-making power
		Additional burden of household chores
		Lack of mobility
B	Economic disparities	Financial dependency on males of the family
		Low control over income (both farm and non-farm)
		Lack of access to resources
C	Technical disparities	Lack of training/skill development opportunities
		Lack of access to extension and advisory services
		Poor technical knowledge on citrus farming

### Gender disparities and its effect on agricultural technology adoption by women farmers

**Figure 8:**
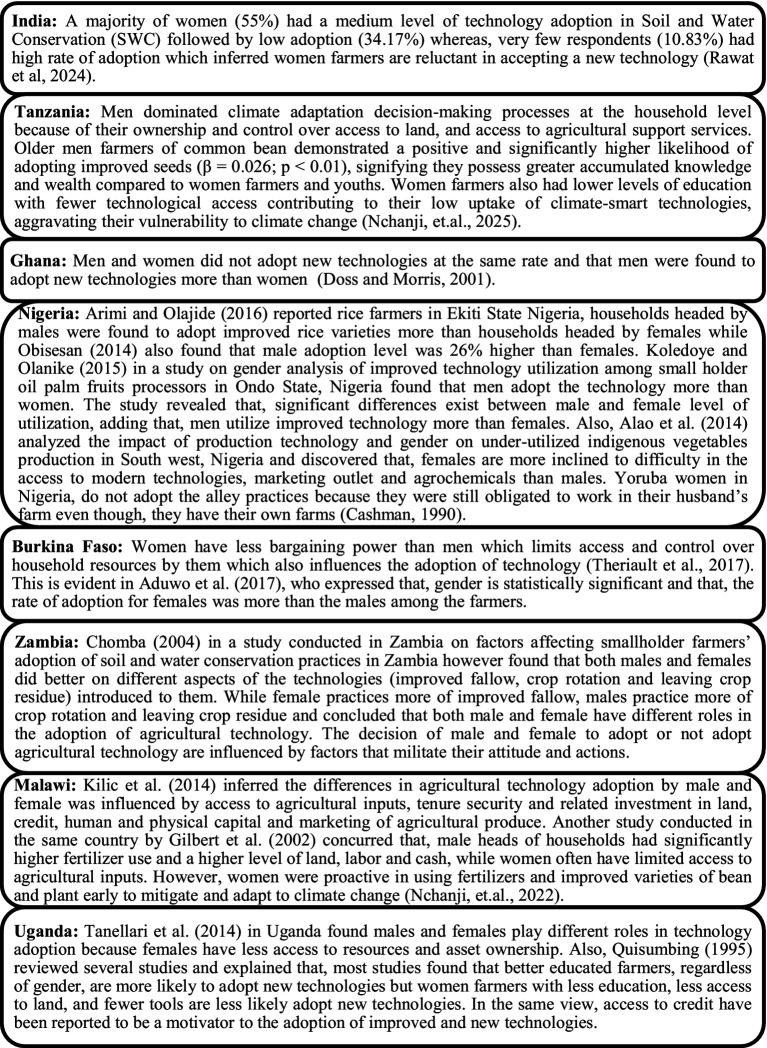
Review of studies on gender disparities affecting agricultural technology adoption by women.

Gender disparities significantly impact women’s adoption of agricultural technologies, often leading to lower productivity and limited participation in farming advancements. Women farmers frequently face barriers in accessing essential resources like land, credit, inputs, and extension services. Traditional gender roles and societal expectations can restrict women’s access to information, training, and decision-making power regarding technology adoption. Women often shoulder a disproportionate burden of household and caregiving responsibilities, limiting their time and capacity to engage in new technologies. Agricultural technologies are often designed without considering the specific needs and constraints of women farmers, hindering their adoption. Women farmers often receive fewer visits from extension agents, limiting their access to crucial information and support. High input costs, limited access to credit, and lack of collateral can prevent women from adopting new technologies. Hence, a cross-country visualization was done of 996 studies obtained after a PRISMA search, pertaining to gender disparities and technology adoption by women. Studies from India, Tanzania, Nigeria and other countries of Africa (Burkina Faso, Ghana, Uganda, Zambia, and Malawi) were selected as these countries had maximum studies on women and citriculture as per bibliographic analysis illustrated in Figure 6. With regard to this fact, research studies conducted in these specific countries on gender gaps affecting technology adoption were selected for the general review. As specific studies on adoption of citriculture technologies by women were not available hence, adoption of agricultural technologies by women was analyzed (Figure 8) from these countries.

The macroanalysis of cross-country studies on gender gaps affecting agricultural technology adoption revealed (Figure 8) that gender-based constraints contribute to lower adoption rates of improved seeds, fertilizers, and other technologies. Limited access to resources and technology leads to lower agricultural yields and reduced income for women farmers. Gender disparities in agricultural technology adoption exacerbate existing inequalities in wealth, income, and access to opportunities. In the long run, women farmers are more vulnerable to climate change impacts and food insecurity due to their limited access to adaptation technologies (Rawat et al., 2024; Nchanji et al., 2025; Doss and Morris, 2001; Arimi and Olajide, 2016; Obisesan, 2014; Koledoye and Olanike, 2015; Alao et al., 2014; Cashman, 1990; Theriault et al., 2017; Aduwo et al., 2017; Chomba, 2004; Kilic et al., 2014; Gilbert et al., 2002; Nchanji et al., 2022; Tanellari et al., 2014; Quisumbing, 1995).

### Status of ‘women in citriculture’ in the ‘society’: microanalysis

#### Resources profile

The microanalysis showed that the majority of women citrus growers (65%) were middle aged (30–50 years) and 98% of them had no land holding in their own name (Table 5), working in the orchards of their husbands or fathers, which were also mostly (65%) 2–4 ha in size. Only 2% had land in their own name. The *t*-values from an independent sample *t*-test were found to be significant at 1 and 5% levels of significance, indicating the means were significantly different.

**Table 5:** Socioeconomic profile of respondents of the microanalysis (*n* = 300).

S. No.	Socioeconomic Parameters	Categories	Percent (%) of respondents	*t*- value
1	Age (years)	<30	7	2.54*
		30–50	65	
		>50	28	
2	Land holding (ha)	Yes	2	3.94**
		No	98	
3	Size of citrus orchard (ha) (irrespective of own name or not)	<2	30	2.01*
		02–04	65	
		>4	5	
4	Education	Less than or up to class 12	94	3.01**
		Up to graduation	5	
		Up to postgraduation and above	1	
5	Primary engagement	Only citrus farming/processing	9	1.98*
		Citrus farming/processing and household chores	77	
		Additional non-farm jobs and household chores	14	
6.	Access to paid or unpaid farm work	Paid	35	2.05*
		Unpaid	65	

Level of significance at *5 and **1%.

This revealed the stark difference in access to resources and inputs between men and women in citriculture. The women were less educated, with 94% being schooled up to class 12, only 5% graduates, and only 1 woman had postgraduation degree. Hence, most middle-aged, less educated ones (77%) were contributing their labor in citrus farming/processing and household chores. A handful (14%) of them were working in some additional non-farm jobs (mostly part-time in nature) after their little bit of farming/processing activities, but they, too, were burdened with household chores. Only nine of them had managed to focus on farming/processing work because they had large joint families where the household chores were mostly handled by other women members who stayed at home. However, even they confessed that they had to dedicate a significant amount of time to caring for their children, helping other women members with household chores, and taking care of the other family members, especially the elderly and men who had less time available. About 35% were agricultural laborers who got paid for their engagement in farm activities, while the remaining 65% were doing unpaid labor on their family farms. Findings are similar to Raney et al. (2011), who reported that women were overrepresented in unpaid, seasonal, and part-time work, often being paid less than men, for the same work. Women’s engagements are mostly restricted to unpaid labor, be it in family farming or household caregiving, which leaves them with less time for self-development activities. Similar findings were reported by Nchanji et al. (2021) among women bean producers of Kenya.

#### Agency profile

The indicators of domain agency depict the control or command a woman has in the decision-making process of the household. In citrus farm families, approximately 62% of women reported being engaged with citrus farming activities, such as weeding, intercultural operations in the orchard, harvesting, grading, and sorting, while 27% women were involved in processing activities of citrus, preparing value-added products out of citrus. Only 11% of women took their produce to market to sell it themselves. The data makes it evident that marketing is still looked upon as an activity where women should not venture, as it involves direct interaction with unknown people, dealing with money, and traveling, too. Patriarchal notions like women are meant to be confined to households and should not handle money are the basis of such an attitude toward women. The findings are similar to those of a study conducted on women farmers of Rajasthan, India by Chayal and Dhaka (2010), where women were engaged more in drudgery-prone field activities than marketing.

None of the respondents reported having full control over farm or non-farm income. It was either the men members having full control over farm (54%) and non-farm (32%) income, or the man and woman jointly (25 and 18%, respectively) deciding on them. A significant 21 and 50% of women stated that their husbands/fathers consult them, but the final decision is taken by the men members related to the use of farm and non-farm income, respectively, for meeting household expenses. In decisions related to healthcare or the education of children, women had slightly more decision-making power or say in the household because 23% women reported that they make the decision fully, and only financial expenses are paid by the father. About 70% stated that they jointly decide, only 7% reported that the men members consult, but the final decision is taken by the men. None of them reported that males had full decision-making power with regard to the education or healthcare of children. This shows that even when women lack financial control, they are often consulted on decisions related to family wellbeing and caregiving, especially concerning children, and men rely on their decision-making capabilities in these matters. Findings are in consonance with Rosada (2016) and Herawati et al. (2021).

Related to domestic violence, only 8% women reported that they did not face any kind of violence in their households. The majority (approximately 77%) reported that they had witnessed domestic violence in some form or the other at some point of time in their households, while 15% stated that they were active victims of domestic violence. In terms of mobility, 52% reported that they were not independent in moving and had to depend on someone or seek permission for moving out of the house almost always, 46% reported that they had to seek permission or depend on other members sometimes. Only 2% women reported that they enjoyed full freedom of mobility. These women were mostly associated with processing and marketing activities, which required them to move out often. The findings are similar to those of Wood et al. (2024), Frankenthal and Dutta (2021), and Parveen (2007) from Saskatchewan, India, and Pakistan.

#### Achievements profile

The microanalysis revealed that 65% women respondents were part of either SHGs, FIGs, or FPOs, but none were part of any FPC. They also stated that none of them were representatives/workers of political parties, hence political representation was nil in the sample. The findings are similar to those from Australia, where rural women were largely invisible in public areas of influence (Alston, 2003) due to the agricultural agenda being largely framed around a masculinist position, and women, even if elected to a position, remain merely as shadowy presences of outraged silence. Women’s underrepresentation in the political arena is mostly due to the gendered structure of society.

### Gender disparities affecting citrus-based technology adoption: cues from agency and achievement profile

The gender disparities faced by women citrus growers were found to affect the extent of scientific technology adoption of citrus. Scientific citriculture, which results in better yield and productivity of citrus, is technology intensive. However, the microanalysis revealed most women (32%) were ignorant of the advanced technologies in citrus production/processing and felt underconfident in expressing their technical expertise (Table 6). However, they were contributing to major operations, including weeding, spraying of pesticides and insecticides, intercultural operations, fertilizer application, harvesting, fresh juice extraction, and selling fruits in local markets. Almost none, except three respondents, had received some skill development training in citrus. So, it became evident that without adequate technical expertise, they were contributing to this sector for decades. As a result, none of them had complete control over the decision-making process of citrus-based technology adoption in their farms or processing ventures. Only 15% of them decided jointly, while 24% gave their inputs, but the final decision was taken by the men family members. A surprising 61% of women citrus growers were not consulted by their men family members when making decisions on technology adoption in their citrus ventures. Only 15% had some kind of contact with extension professionals of the state agriculture or horticulture department or private consultancies. However, that contact too was dependent on when the professionals visited their villages.

**Table 6:** Gender disparities and their effect on citrus-based technology adoption by women in different activities of the citrus value chain (n = 300).

Technology Adoption Parameters	Presence of sound technical know-how of citrus farming/processing		Say in the household in deciding which technology to adopt in the farming/processing of citrus				Training or facilitation received from horticulture/agriculture extension professionals for involvement in the citrus value chain		Do you have any access to extension or any kind of citrus advisory services?	
Categories with scores	Yes = 1	No = 0	Yes I decide = 3	We jointly decide with husband/father = 2	I give inputs and my husband/father takes final decision = 1	My husband/father does not consult me = 0	Yes = 1	No = 0	Yes = 1	No = 0
% of respondents	32	68	0	15	24	61	3	97	15	85
	Maximum obtainable technology adoption score = 6 (if any respondent scores 1 + 3 + 1 + 1 in the above 4 parameters)									
	Technology adoption score (0)			Technology adoption score (1–3)			Technology adoption score (4–6)			
	42%			38%			20%			

The Technology Adoption Scores were calculated by linear addition of scores obtained on four parameters of technology adoption questions (Table 6). The maximum obtainable score being 6, the majority (42%) of women scored 0, 38% scored 1–3, and only 20% scored 4–6. The scores represented their extent of contribution in the technology adoption process in their citrus ventures. However, as is evident, a large section of women, though contributing toward citrus farming in different ways, played no to a very insignificant role in technology adoption.

If half of the population involved in a venture does not have any say in the technology adoption process, it clearly presents the gender gap, which ultimately triggers the technology gap in any sector. Lack of knowledge, skill, and decision-making power of women affects the overall technology adoption process of any sector of agriculture, and this finding is in alignment with that of Shahbaz et al. (2022), Ragasa (2012), Kassie et al. (2020), Doss and Morris (2000), and Mwinuka and Hyera (2022). It was reflected vividly through the Technology Adoption Scores.

The relation between socioeconomic parameters and technology adoption parameters were also analyzed (Table 7) through a chi-squared test, which revealed that age of the respondent, size of land holding possessed by the family, size of citrus orchard possessed and education of the respondent were significantly associated (χ^2^value significant at 0.05 level of significance) with citrus based technology adoption by women citrus growers. Women of younger age, larger land holdings, orchards, and higher education tended to have higher adoption scores. The findings are similar to technology adoption studies done in vegetable farming and livestock farming, too (Sanya et al., 2025; Rezvanfar, 2007; Mohanty et al., 2013; Zanu et al., 2012).

**Table 7:** Association between socioeconomic parameters and citrus-based technology adoption.

S. No.	Socioeconomic parameters	Chi-squared value (*χ*^2^)
1.	Age (years)	12.06*
2.	Land holding (ha)	10.05*
3.	Size of citrus orchard (ha)	5.35*
4.	Education	10.03*
5.	Primary engagement	1.09

*Level of significance at 5%.

### Correlating macro and microanalysis: bridging science and society

Macroanalysis revealed the primary gender disparities (Table 4) that existed in citrus farming, processing, or, in other words, citrus-based farm households. These gender issues were ranked severity-wise by the respondents of the microanalysis. The results were subjected to a Friedman ranking. It was found that lack of access to basic inputs and resources was the major challenge, and it was adjudged as most severe by almost 98% of respondents (Table 8).

**Table 8:** Severity of gender disparities.

Sl. no.	Items/aspects	Friedman mean rank	Mean %	Overall rank
A. Socio-personal disparities				
1.	Sole responsibility of taking care of family and children	2.11	45	X
2.	Low decision-making power	3.08	60	VIII
3.	Additional burden of household chores	2.81	55	IX
4.	Lack of mobility	3.46	62	VII
B. Economic disparities				
5.	Financial dependency on men of the family	5.90	92	II
6.	Low control over income (both farm and non-farm)	5.18	86	III
7.	Lack of access to resources	5.01	82	IV
C. Technical disparities				
8.	Lack of training/skill development opportunities	5.00	81	V
9.	Lack of access to extension and advisory services	4.89	76	VI
10.	Poor technical knowledge of citrus farming	5.97	95	I

About 95% respondents reported that lack of technical knowledge (5.97) and 92% reported financial dependency (5.90) on men family members were the two most severe gender disparities. Low control over income (86%), Lack of access to resources (82%), Lack of Training (81%), and Lack of access to extension services (76%) were the next big constraints. Lack of mobility (62%), lack of freedom (60%), household chores (55%), and care of families and children (45%) were some other factors that were reported to restrain women. Hence, the gender issues that existed globally in citriculture since 1929 were validated through the microanalysis in 2024. The longitudinal and cross-sectional differences impacted women very little because, irrespective of any part of the world or time, women were still the unsung part of citrus stories.

The results from the independent sample t-test presented in Table 9 revealed that there was no significant difference in the degree of economic and technical gender disparities faced by the technically sound and not-so-sound respondents. However, there was a significant difference in the degree of socio-personal constraints faced by the two categories of respondents, as the *t*-value (2.02) was found to be significant at the 0.05 level. It can be inferred from the results that the low technical know-how of the respondents can be attributed to the higher degree of socio-personal disparities that they face.

**Table 9:** Category of gender disparities influencing technical knowledge on citrus.

Sl. no.	Category of gender disparities	Presence of sound technical know-how of citrus farming and processing (mean %)	Lack of sound technical know-how of citrus farming and processing (mean %)	*t*-value
A	Socio-personal disparities	37.74	62.26	2.02*
B	Economic disparities	58.93	41.07	1.29^NS^
C	Technical disparities	57.12	42.88	1.77^NS^

NS = non-significant; Level of significance at *5 and **1%.

### Bridging science and society through a gender transformative approach at the microlevel

The empirical findings of the study have presented the “macro” and “micro” picture of women citrus growers in “science” and “society.” The macrolevel findings of science bear similarities and relevance even to this date in our microlevel society, as evident through the correlation. The transformation of women citrus growers in this study can be facilitated through the implementation of women empowerment programs by the government or other developmental agencies. However, to break the gender barriers from the root level and empower the women farmers would require adopting an approach that deeply diagnoses the gender dynamics of the society and then changes the local level structural gender barriers, which would automatically empower women with greater mobility, more access to resources, more freedom, and control over financial resources. To break gender disparities at the local level, that is, within households and communities, the Gender Transformative (GT) approaches can be utilized. That is a theoretical framework (science) to deal with a societal disparity, or in other words, science reaching out to society.

A GT approach refers to an approach in development or research and development (R&D) that is intentionally oriented, facilitated, and applied with the aim of examining, challenging, and transforming the underlying causes of gender inequality. These causes are rooted in social structures, including the gender norms that underpin imbalances in gender power dynamics, roles, and relations. The guiding theory is that by transforming underlying drivers, the GT approach breaks the cycle of gender barriers and constraints being created and perpetuated over time (McDougall et al., 2023). GT methods describe the procedures and practices by which the GT approach is operationalized. There are two types of GT methods that can be applied at the microlevel or at the local level. Formative GT methods can be used during the scoping and design stage of the program. They generate a diagnostic understanding of (intersectional) gender dynamics (interactions, relations, and patterns of behavior) and, specifically, of underlying gender barriers that create those dynamics in particular contexts. They may assess, for example, the gender division of labor, decision-making, and distribution of benefits or burdens, and seek to identify the underlying factors (e.g., norms and belief systems) that drive these. Their primary focus is on generating data to inform the design of catalytic GT strategies (next stage) and to inform overall project design. Catalytic GT methods are then designed to spark and build change during the implementation stages of a project or program. Their primary focus is on catalyzing change in local-scale structural gender barriers, such as constraining gender norms, to lay the foundations for greater equality. Catalytic methods, as used here, are not only about developing interest or commitment for change in support of equality among people of all genders within households, groups, and the wider community, but also operationalizing (piloting, actioning) ideas or strategies for change.

The GT approaches have been applied successfully in various women empowerment programs. Macnaughton et al. (2016) reported the integration of empowerment and intersectionality as a Gender Transformative approach for rural indigenous fishing communities in the Bolivian Amazon through the Pecespara la vida (PPV) project (“Fish for Life”). Escobar et al. (2017) indicated the Papa Andina initiative, as implemented by the International Potato Center (CIP) in Peru, as a Gender Transformative approach. Papa Andina supported peasant women producers in participating in new processes of institutional, organizational, commercial, technological, and social innovation. Toward the end of the program, Papa Andina became more responsive to resource-poor women, acknowledging gender dynamics within some coalitions and gender gaps within innovation processes, particularly in terms of gendered access to and control over resources and assets for native potato value chains. Mobility and participation in the decision-making processes allowed women and men to gain self-confidence and respect inside the Papa Andina project, their communities, and their own households.

### Strategies for empowering women citrus farmers

**Figure 9:**
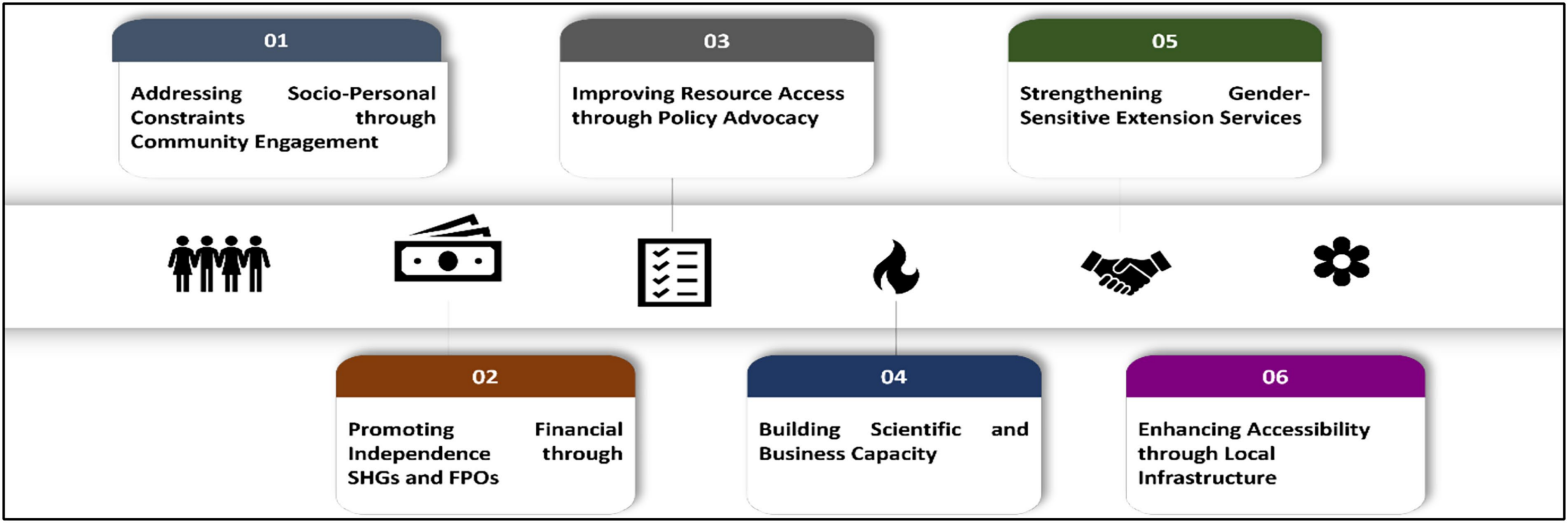
Strategies for empowering women citrus farmers.

Formulating and implementing strategies to bridge the existing gender gap in the citrus sector is the need of the hour. The following strategies, ranked by the severity of gender disparities (as mentioned in Table 8), are suggested to reduce the gender discrepancies found in the study. These strategies provide focused and effective interventions by addressing the socio-personal, economic, and technical inequalities that women in citrus farming confront (Figure 9).

- Addressing socio-personal constraints through community engagement

Implementing time-saving technologies can reduce the strain of family chores, freeing up more time for women to engage in citrus farming. Additionally, running community awareness programs can encourage shared household responsibilities and promote gender equity in decision-making.

- Promoting financial independence through SHGs and FPOs

Although this study involved women citrus growers who were members of Self-Help Groups (SHGs), control of their husbands or men of their families over their earnings from SHGs curbed their financial freedom. In this case, the men members of households can be made a part of the same SHGs so that both men and women farmers of a farm household can have some disposable income, access to loans, etc. Participation in Farmer Producer Organizations (FPOs), Farmer Interest Groups (FIGs), and Self-Help Groups (SHGs) can give them access to low-interest loans and microfinance for citrus farming inputs, reducing the women’s reliance on the man of the household and addressing economic inequality.

- Improving resource access through policy advocacy

Encouraging gender-sensitive policies to guarantee women farmers’ fair access to financial services and agricultural inputs through targeted subsidies; utilizing programs, such as the *Namo Drone Didi Initiative* and the *Mahila Kisan Sashaktikaran Pariyojana* (MKSP), to increase women’s involvement in agriculture in India. Inequalities in resource access can be addressed by facilitating access to instruments appropriate for citrus cultivation, such as precision pruning tools and battery-operated sprayers, which can enhance their independence and productivity.

- Building scientific and business capacity

By providing business planning and computer literacy workshops, as well as training programs on scientific citrus farming that are targeted at women and cover a varied range of topics like soil health, plant nutrition, and best practices, we can help close the technological gap in agriculture by enhancing technical knowledge and decision-making abilities.

- Strengthening gender-sensitive extension services

Encouraging the use of mobile-based platforms and easily navigable expert systems to improve access to timely information and close gaps in mobility and extension services; encouraging the participation of women extension workers from state agricultural universities, public and private research institutes, and non-governmental organizations to provide context-specific advisory services on citrus farming.

- Enhancing accessibility through local infrastructure

Supporting women with limited mobility and family care responsibilities by promoting community-based transportation options and creating local resource hubs connected to government agencies, private input dealers, and non-governmental organizations to offer market access, training, and advice.

By increasing citrus-cultivating women farmers’ access to resources, information, technology, and support networks, these policies collectively seek to empower women farmers, advance gender parity, and strengthen their position in sustainable citrus production.

## Conclusion

The results of the study indicate that the quantum of research done on women in citriculture requires a major boost. Stories of women need to be told, documented, researched upon and actionable policies formulated as part of addressing the gender disparities that exist. As socio-personal gender disparities and socio-economic parameters have a strong association with the technical knowledge and technology adoption parameters, emphasis should be given to addressing the socio-personal gender issues as a priority through social security, education, and capacity-building interventions. Assessment of women’s role, contribution, level of participation, and decision-making is essential in citrus farming. Women empowerment is considered a ‘prerequisite’ to achieving global food security, and country-specific gender intervention frameworks are necessary to overcome gender gaps in agriculture. Gender-sensitive strategies for financial, legal, economic, and social empowerment of women farmers should be the priorities of policymakers to achieve the targets of Sustainable Development Goals of the UN set for every country. Both gender equality and equity are the crux of social development globally. At the foundation of this step lies the essential need to “tell” their stories, acknowledge the gender gaps, and act to bridge the gaps.

## Data Availability

The original contributions presented in the study are included in the article/supplementary material, further inquiries can be directed to the corresponding author.

## References

[ref1] Adesiyan F. O. Adesiyan A. T. Yesufu A. O. Akinola O. (2015). Productivity differentials of improved rice variety adoption among male and female headed households in Ekiti state, Nigeria. Gender, Agric., Environ., Technol., 315–327.

[ref9001] Aduwo O. E. Aransiola J. O. Ikuteyijo L. O. Alao O. T. Deji O. F. Ayinde J. O.. (2017). Gender differences in agricultural technology adoption in developing countries: a systematic review. Afri-Veg Forum. 1238, 227–238. doi: 10.17660/ActaHortic.2019.1238.24

[ref2] Akhtar S. Palaniappan G. Ashraf E. Shaheen A. Sadaf S. (2024) Women in Citrus value chain: A challenge and opportunity. National Citrus Conference, March 5, 2024, Pakistan.

[ref3] Alao O. T. Adebooye O. C. Deji O. F. Idris-Adeniyi K. M. Agbola O. Busari O. A. (2014). Analysis of the impact of production technology and gender on under-utilized indigenous vegetable production in South-Western Nigeria. Afr. J. Sci. Technol. Innov. Dev. 6, 51–59. doi: 10.1080/20421338.2014.931741

[ref4] Alston M. (2003). Women's representation in an Australian rural context. Sociol. Ruralis 43, 474–487. doi: 10.1046/j.1467-9523.2003.00256.x

[ref9002] Arimi K. Olajide B. R. (2016). Comparative analysis of male and female adopters of improved rice production technology in Ogun and Ekiti states, Nigeria. Int. J. Agric. Resour. Gov. Ecol. 12, 246–261. doi: 10.1504/IJARGE.2016.078305

[ref5] Babu S. C. Gajanan S. N. (2022). “Effects of technology adoption and gender of household head: the issue, its importance in food security—application of Cramer's V and phi coefficient” in Food security, poverty and nutrition policy analysis. eds. Babu S. C. Gajanan S. N.. Third ed (Elsevier, Cambridge: Academic Press), 105–133.

[ref7] Cashman K. (1990) A grounded theory describing factors in the adoption process of the alley farming technology by Yoruba women in Nigeria (Iowa State University digital repository @ Iowa State University). Available online at: https://dr.lib.iastate.edu/entities/publication/2ea8cac4-cd74-4df1-bf80-6b4a6a233cbc (Accessed 15 June, 2025).

[ref8] Chayal K. Dhaka B. L. (2010). Analysis of role performance of women in farm activities. Age 30:30. Available online at: https://api.seea.org.in/uploads/pdf/v10224.pdf (Accessed 25 August, 2025).

[ref9] Chomba N.G. (2004) Factors affecting smallholder farmers' adoption of soil and water conservation practices in Zambia. Thesis submitted to the Department of Agricultural Economics (Michigan State University).

[ref10] Doss C. R. (2018). Women and agricultural productivity: reframing the issues. Develop. Policy Rev. 36, 35–50. doi: 10.1111/dpr.12243, PMID: 29263585 PMC5726380

[ref11] Doss C. R. Morris M. L. (2000). How does gender affect the adoption of agricultural innovations? The case of improved maize technology in Ghana. Agric. Econ. 25, 27–39. doi: 10.1111/j.1574-0862.2001.tb00233.x

[ref12] Doss C. R. Morris M. L. (2001). How does gender affect the adoption of agricultural innovations? The case of improved maize technology in Ghana. Agric. Econ. 25, 27–39. doi: 10.1016/S01695150(00)00096-7

[ref13] Escobar S. S. Odame H. H. Tegbaru A. (2017). “Gender transformative approaches in agricultural innovation: the case of the papa Andina initiative in Peru” in Sustainable intensification in smallholder agriculture: An integrated systems research approach. eds. Öborn I. Vanlauwe B. Phillips M. Thomas R. Brooijmans W. Atta-Krah K. (Routledge, Oxfordshire, England: Routledge), 304–316.

[ref14] FAO (2009). High level expert forum - how to feed the world in 2050. Office of the Director, agricultural development economics division economic and social development department. Rome, Italy: Vialedelle Terme di Caracalla.

[ref15] Frankenthal I. Dutta D. (2021) Risk factors for gender-based violence. Oxfam America: The case of Indian agriculture

[ref16] Gilbert R. A. Sakala W. D. Benson T. D. (2002). Gender analysis of a nationwide cropping system trial survey in Malawi. Afr. Stud. Q. 6, 1–2. Available at: https://journals.flvc.org/ASQ/article/view/136406/140967 (Accessed 20 September, 2025).

[ref17] github. (n.d.). Available online at: https://saylordotorg.github.io/text_principles-of-sociological-inquiry-qualitative-and-quantitative-methods/s05-01-micro-meso-and-macro-approache.html (Accessed November 30, 2024)

[ref18] Gonzatto P.M. Santos S.J. (2023). Introductory chapter: world Citrus production and research. London, United Kingdom: Intech Open

[ref19] Herawati T. Simanjuntak M. Kumalasari B. (2021). Investigating the quality of life on farmer family: roles of gender relations, economic pressure, financial management, and livelihood strategies. J. Fam. Sci. 6, 37–52. doi: 10.29244/jfs.v6i1.35796

[ref20] Kabeer N. (1999). Resources, agency, achievements: reflections on the measurement of women’s empowerment. Dev. Change 30, 435–464. doi: 10.1111/1467-7660.00125

[ref21] Kabeer N. (2005). Gender equality and women’s empowerment: a critical analysis of the third millennium development goal. Gend. Dev. 13, 13–24. doi: 10.1080/13552070512331332273

[ref22] Kassie M. Fisher M. Muricho G. Diiro G. (2020). Women’s empowerment boosts the gains in dietary diversity from agricultural technology adoption in rural Kenya. Food Policy 95:101957. doi: 10.1016/j.foodpol.2020.101957

[ref23] Kilic T. Palacios-Lopez A. Goldstein M. (2014). Caught in a productivity trap: a distributional perspective on gender differences in Malawian agriculture. World Dev. 70, 416–463. doi: 10.1016/j.worlddev.

[ref24] Kilmanun J. C. Warman R. Burhansyah R. (2023). Contribution of women labor to orange farmers income in tebas sub-district sambas regency. IOP Conf. Ser. Earth Environ. Sci. 1153:012037. doi: 10.1088/1755-1315/1153/1/012037

[ref25] Koledoye G. Olanike D. (2015). Gender analysis of technology utilisation among small scale oil palm fruits processors in Ondo State, Nigeria. Acta Agron. 64, 36–47. doi: 10.15446/acag.v64n1.42908

[ref26] Liberati A. Altman D. G. Tetzlaff J. Mulrow C. Gøtzsche P. C. Ioannidis J. P. A.. (2009). The PRISMA statement for reporting systematic reviews and meta-analyses of studies that evaluate health care interventions: explanation and elaboration. PLoS Med. 6, 1–28. doi: 10.1371/journal.pmed.1000100, PMID: 19621070 PMC2707010

[ref27] Macnaughton A. E. Rainville T. K. Méndez C. I. C. Ward E. M. Wojciechowski J. M. Carolsfeld J. (2016). “Gender transformative approaches with socially and environmentally vulnerable groups: indigenous fishers of the Bolivian Amazon” in Transforming gender and food security in the global south. eds. Njuki J. Parkins J. R. Kaler A. (Routledge, Oxfordshire, England: Routledge), 217–240.

[ref28] Maier A. (2024). A woman’s world: A history of female labor in Citrus packinghouses. Sweet and sour Citrus. Accessed on 5th may, 2024. Available online at: https://sweetandsourcitrus.org/a-womans-world-a-history-of-female-labor-in-citrus-packinghouses/ (Accessed 9 December, 2024).

[ref29] Manenzhe T. D. (2021). The constrains affecting competitiveness of emerging household Citrus farmers in Mpumalanga Province, South Africa. Eur. J. Agric. Food Sci. 3, 33–39. doi: 10.24018/ejfood.2021.3.5.343

[ref30] McBane M. (2001). The house that lemons built: Race, ethnicity, gender, citizenship and the creation of a citrus empire, 1893–1919. Los Angeles: University of California.

[ref31] McDougall C. Elias M. Zwanck D. Diop K. Simao J. Galiè A.. (2023). Fostering gender-transformative change for equality in food systems: A review of methods and strategies at multiple levels. CGIAR GENDER impact platform working paper #015. Nairobi, Kenya: CGIAR GENDER Impact Platform.

[ref32] Meinzen-Dick R. Rubin D. Elias M. Myers E. (2019). Women’s empowerment in agriculture: Lessons from qualitative research. IFPRI discussion paper 01868: International Food Policy Research Institute (Washington DC, USA: IFPRI).

[ref9004] Ministry of Agriculture and Farmers Welfare. (2023). 2021-22 (First Advance Estimates) of Area and Production of Horticulture Crops. Ministry of Agriculture & Farmers Welfare, Government of India.

[ref33] Mohanty A. K. Lepch B. Kumar A. (2013). Constraints analysis in adoption of vegetable production technologies for livelihood perspective of tribal farmers in North Sikkim. Indian Res. J. Ext. Educ. 13, 51–56. Available at: http://www.seea.org.in/vol13-2-2013/10.pdf (Accessed 28 August, 2025).

[ref34] Mwinuka L. Hyera E. O. (2022). Effect of socio-cultural factors on gendered decision-making in the adoption of improved maize storage technologies. Cogent Food Agric. 8:2132849. doi: 10.1080/23311932.2022.2132849

[ref35] Nchanji E. B. Kabuli H. Nyamolo V. O. Cosmas L. Chisale V. Matumba A. (2022). Gender differences in climate-smart adaptation practices amongst bean-producing farmers in Malawi: the case of Linthipe extension planning area. Front. Sustain. Food Syst. 6:1001152. doi: 10.3389/fsufs.2022.1001152

[ref36] Nchanji E. B. Mutua M. Odhiambo C. Nchanji Y. K. Karanja D. (2021). Deconstructing leisure time and workload: case of women bean producers in Kenya. Agric. Food Secur. 10:12. doi: 10.1186/s40066-021-00286-w

[ref37] Nchanji E. B. Ndunguru A. Kabungo C. Katunzi A. Nyamolo V. Ouya F. O.. (2025). Assessing gender disparities in farmers’ access and use of climate-smart agriculture in southern Tanzania. Discover Sustain. 6, 1–15. doi: 10.1007/s43621-025-01150-8 PMC12037644 40308685

[ref38] Obisesan A. (2014). Gender differences in technology adoption and welfare impact among Nigerian farming households. Munich: Munich Personal RePEc Archive. Available online at: http://mpra.ub.uni-muebchen.de58920 (Accessed 25 June, 2025).

[ref39] Parveen S. (2007). Domestic violence against farm women in Bangladesh: causes and consequences. Pak. J. Womens Stud. Alam-e-Niswan 14, 103–117. Available online at: https://openurl.ebsco.com/EPDB%3Agcd%3A2%3A22142994/detailv2?sid=ebsco%3Aplink%3Ascholar&id=ebsco%3Agcd%3A31566440&crl=c&link_origin=scholar.google.com (Accessed 18 September, 2025).

[ref40] Quisumbing A. R. (1995). Gender differences in agricultural productivity: A survey of empirical evidence. Esp discussion paper series no.36, Education and Social Policy Department. Washington, DC, USA: The World Bank.

[ref41] Ragasa C. (2012) Gender and institutional dimensions of agricultural technology adoption: A review of literature and synthesis of 35 case studies 2012 Conference, Brazil: International Association of Agricultural Economists. 126747. doi: 10.22004/ag.econ.126747

[ref42] Rana H. (2024). Examining the ‘feminization of agriculture’ in a mixed-farming system in Sindhuli District, Nepal. HIMALAYA, 43, 133–148. doi: 10.2218/himalaya.2024.8123

[ref43] Raney T. Anríquez G. Croppenstedt A. Gerosa S. Lowder S. K. Matuschke I.. (2011). The role of women in agriculture. Food and Agriculture Organization of the United Nations, Agricultural Development Economics Division (ESA). doi: 10.22004/ag.econ.289018

[ref44] Rawat I. Jakhar P. Jinger D. Sharma G. Kumar M. Jhajhria A.. (2024). Farm women participation in natural resource conservation: technology adoption study in semi-arid regions of India. Bhartiya Krishi Anusandhan Patrika. 39, 66–73. doi: 10.18805/BKAP710

[ref45] Rezvanfar A. (2007). Communication and socio-personal factors influencing adoption of dairy farming technologies amongst livestock farmers. Livest. Res. Rural. Dev. 19:33. Available online at: https://lrrd.cipav.org.co/lrrd19/3/rezv19033.htm (Accessed 20 September, 2025).

[ref46] Rosada I. (2016). A review on multi-roles of women and their influence on the change of functional structure in the farmer's household. Agric. Agric. Sci. Procedia 9, 47–53. doi: 10.1016/j.aaspro.2016.02.122

[ref47] Sahu A. Kishore K. J. C. J. Srivastava S. K. Mohapatra L. (2018). Understanding gender dynamics in fruit cultivation under Indian condition. Int. J. Curr. Microbiol. App. Sci. 7, 1923–1933. doi: 10.20546/ijcmas.2018.710.221

[ref9003] Sahu A. Seth T. Sahoo L. Das L. (2023). Enhancing farm women’s employment and income through fruit crops-based farming systems. In: Tanuja S. Kumari V. Das L. Kumar N. Devi M. (2023). Agripreneurship through Farming System: a viable techno-economical approach for women empowerment (E-book) Page 130. Hyderabad: ICAR-Central Institute for Women in Agriculture & MANAGE. 129. Available at: https://agritech.tnau.ac.in/pdf/final%20compendium_240320_150922.pdf (Accessed 21 September, 2025).

[ref48] Sanya L. N. Kyazze F. B. Kweyu Lutomia C. Mukhwami J. T. Kakuru M. Shimali F.. (2025). Determinants of women’s empowerment in the context of sustained adoption of improved crop varieties in rural eastern Uganda. CABI Agric. Biosci. 6:0058. doi: 10.1079/ab.2025.0058

[ref49] Sarker M. N. I. Barman S. C. Islam M. Islam R. Chakma A. S. (2017). Role of lemon (*Citrus limon*) production on livelihoods of rural people in Bangladesh. J. Agric. Econ. Rural Dev. 2, 167–175. Available online at: https://www.researchgate.net/publication/317546288_Role_of_lemon_Citrus_limon_production_on_livelihoods_of_rural_people_in_Bangladesh (Accessed 21 September, 2025).

[ref50] Shahbaz P. Ul Haq S. Abbas A. Batool Z. Alotaibi B. A. Nayak R. K. (2022). Adoption of climate smart agricultural practices through women involvement in decision making process: exploring the role of empowerment and innovativeness. Agriculture 12:1161. doi: 10.3390/agriculture12081161

[ref51] Tanellari E. Kostandini G. Bonabana-Wabbi W. Murray A. (2014). Gender impacts on adoption of new technologies: the case of improved groundnut varieties in Uganda. Afr. J. Agric. Resour. Econ. 9, 300–308. doi: 10.22004/ag.econ.197017

[ref52] Theriault V. Smale M. Haider H. (2017). How does gender affect sustainable intensification of cereal production in the west African Sahel? Evidence from Burkina Faso. World Dev. 92, 177–191. doi: 10.1016/j.worlddev.2016.12.003, PMID: 28373743 PMC5268358

[ref53] Velde P.V.D. Stanley V. Stickler M. (2020). Invisible farmers: Why recognizing and supporting women farmers is key to food and nutrition security. Washington DC, USA: World Bank Blogs. Available online at: https://blogs.worldbank.org/en/developmenttalk/invisible-farmers-why-recognizing-and-supporting-women-farmers-key-food-and (Accessed October 30, 2024)

[ref54] Wood K. Giesbrecht C. J. Brooks C. Arisman K. (2024). “I couldn’t leave the farm”: rural women's experiences of intimate partner violence and coercive control. Violence Against Women 31:10778012241279117. doi: 10.1177/10778012241279117, PMID: 39248216 PMC12414102

[ref55] World Bank (2010) Global strategy to improve agricultural and rural statistics. Economic and sector work. Report number 56719-GLB. Washington, D.C.: World Bank Group. Available online at: http://documents.worldbank.org/curated/en/658261468326169733/Global-strategy-to-improve-agricultural-and-rural-statistics (Accessed 13 January, 2025).

[ref56] Zanu H. K. Antwiwaa A. Agyemang C. T. (2012) Factors influencing technology adoption among pig farmers in Ashanti region of Ghana. J. Agri. Tech. 8: 81–82. Available at: https://www.thaiscience.info/journals/Article/IJAT/10841045.pdf (Accessed 21 September, 2025).

